# Quantitative Ultrasound for Evaluation of Tumour Response to Ultrasound-Microbubbles and Hyperthermia

**DOI:** 10.1177/15330338231200993

**Published:** 2023-09-26

**Authors:** Deepa Sharma, Holliday Carter, Lakshmanan Sannachi, Wentao Cui, Anoja Giles, Murtuza Saifuddin, Gregory J. Czarnota

**Affiliations:** 1 Imaging Research and Physical Sciences, Sunnybrook Health Sciences Centre, Toronto, ON, Canada; 2 Department of Radiation Oncology, Sunnybrook Health Sciences Centre, Toronto, ON, Canada; 3 Departments of Medical Biophysics and Radiation Oncology, Faculty of Medicine, University of Toronto, Toronto, ON, Canada

**Keywords:** animal research, biomarker, breast cancer, cancer, cell death

## Abstract

**Objectives:** Prior study has demonstrated the implementation of quantitative ultrasound (QUS) for determining the therapy response in breast tumour patients. Several QUS parameters quantified from the tumour region showed a significant correlation with the patient's clinical and pathological response. In this study, we aim to identify if there exists such a link between QUS parameters and changes in tumour morphology due to combined ultrasound-stimulated microbubbles (USMB) and hyperthermia (HT) using the breast xenograft model (MDA-MB-231). **Method:** Tumours grown in the hind leg of severe combined immuno-deficient mice were treated with permutations of USMB and HT. Ultrasound radiofrequency data were collected using a 25 MHz array transducer, from breast tumour-bearing mice prior and post-24-hour treatment. **Result:** Our result demonstrated an increase in the QUS parameters the mid-band fit and spectral 0-MHz intercept with an increase in HT duration combined with USMB which was found to be reflective of tissue structural changes and cell death detected using haematoxylin and eosin and terminal deoxynucleotidyl transferase dUTP nick end labelling stain. A significant decrease in QUS spectral parameters was observed at an HT duration of 60 minutes, which is possibly due to loss of nuclei by the majority of cells as confirmed using histology analysis. Morphological alterations within the tumour might have contributed to the decrease in backscatter parameters. **Conclusion:** The work here uses the QUS technique to assess the efficacy of cancer therapy and demonstrates that the changes in ultrasound backscatters mirrored changes in tissue morphology.

## Introduction

Cancer is one of the leading causes of death globally. Researchers worldwide are focusing on developing target-specific therapy that can be activated in the tumour region, sparing healthy adjacent tissue and avoiding systemic toxicity.^1,2^ Emphasis is increasing on individual tailoring of treatment based on early response prediction to improve the outcome of cancer therapy.^3-5^ Various tools are used to assess the treatment response in routine practice. The physical examination relies on the tumour size reduction, which can vary from one examiner to another, and may take several days to weeks following the treatment.^6^ Likewise, the use of contrast-enhanced computed tomography and magnetic resonance imaging to evaluate tumour vascularity pre- and post-treatment has also been investigated.^7-11^ However, the risk of exposure to radiation, contrast administration, cost, and time limits their routine uses. Among others are metabolic imaging and ultrasound imaging that can be operator dependent. Despite all these modalities, biopsy remains the most accurate tool to validate the tumour response.^12,13^ However, the biopsy is invasive, painful and may require imaging assistance which makes its use impractical in routine clinical application.^14^

To address the challenge of early detection of tumour response to treatment, non-invasive, cost-effective, reproducible, and operator-independent, image-based portable quantitative ultrasound (QUS) has gained significant attention in the field of oncology and clinical research.^15^ QUS, unlike diagnostic ultrasonography, is not primarily an imaging modality. It is a tool to measure quantitative variables by which tissue characteristics can be assessed.^16-18^ The premise of QUS is a mathematical and statistical calculation of radiofrequency (RF) data usually discarded by diagnostic ultrasound. QUS has the potential for rapid response analysis and thereby can guide physicians for early treatment modification. It has been known that RF backscatters can reflect the phenotypic characteristics of the tumour extracted from ultrasound imaging. These ultrasound backscatters in tissues are affected by their intrinsic properties and reflect in parameters like acoustic impedance, density, shape, and size of the scattering ultrasound waves. These tissue properties undergo alteration in response to a disease or treatment.^19^ Some of the earliest signs of cell death are condensation, segregation, and fragmentation of chromosomes and nucleus. These phenomena generate specific scattering of ultrasound waves. QUS analyses these raw RF data to measure quantitative variables by which one can assess tissue/tumour microenvironment and reflect tissue properties beyond conventional ultrasound. QUS spectral parameters derived from linear regression analysis like the mid-band fit (MBF), spectral 0-MHz intercept (SI), and spectral slope (SS) have been applied in the characterization of various tumours.^20-23^ MBF is found to be related to effective scatterer size, acoustic concentration, and attenuation of scatterer. SI is related to effective scatterer size and acoustic concentration, while SS is related to scatterer size. Several clinical and pre-clinical studies have shown that changes in these spectral parameters are associated with tissue microstructural alterations in response to treatment.^24-32^ In addition to spectral parameters, the analysis of textural characteristics of QUS-based parametric maps using Grey Level Co-occurrence Matrix can provide second-order statistics by determining the patterns of grey-level transitions.^17,33-40^ The texture features can further help distinguish a benign tissue from a malignant based on the homogeneous/heterogeneous patterns of tumours.^41^

In this study, we investigated the sensitivity of QUS spectral parameters as a non-invasive way of identifying cell death following ultrasound-stimulated microbubbles (USMB) and hyperthermia (HT) treatment. QUS data collected were further validated by the results obtained from histopathology analysis. The results demonstrated a statistically significant correlation between ultrasound backscatter intensity related spectral parameters (MBF and SI) with changes in cells and tissue microstructure. The combination of USMB and HT resulted in the highest increase in MBF and SI parameters that were correlated with an increase in cell death detected using haematoxylin and eosin (H&E) and terminal deoxynucleotidyl transferase dUTP nick end labelling (TUNEL) staining. However, no changes were observed in SS parameters in response to treatment.

## Materials and Methods

The reporting of this study conforms to ARRIVE 2.0 guidelines.^42^ Adequate care of the animals were taken following the guidelines of National Research Council (US) Committee for the Update of the Guide for the Care and Use of Laboratory Animals.^43^

### Cell Culture and Animal Model

In this study, MDA-MB-231 breast cancer cell lines were used to grow tumours in the hind leg of female severe combined immunodeficiency (SCID)-B17 mice obtained from Charles River Canada. Cells obtained from the American Type Culture Collections (ATCC, MD, USA) were incubated at 37 °C in 5% CO_2_ in RPMI-1640 medium supplemented with 10% fetal bovine serum (Sigma-Aldrich) and 1% penicillin/streptomycin antibiotics (ThermoFisher Scientific). On reaching 80% confluency, a 0.05% Trypsin–EDTA solution (Wisent BioProducts) was used to trypsinize the cells in preparation for injection. For tumour induction, 5 × 10^6^ cells/100 µL cells were re-suspended in Mg+/Ca+ Dulbecco's phosphate-buffered saline and were injected in the hind limb of the mice. It took about 4–6 weeks of xenografts to grow 5–10 mm and ready for treatment. A mixture of ketamine (100 mg/kg) andxylazine (5 mg/kg) was used to anesthetize animals during the injection of the cells and the therapy procedure. Throughout the experiments, mice were closely monitored by placing the heat lamps and heating pads nearer to prevent irregular body temperature. Each treatment group consisted of 4 or more than 4 animals.

### Ultrasound and Microbubble Treatment

Definity microbubble at a concentration of (3%, v/v) was used for this study. During USMB treatment, tumour-bearing limb was submerged in a 37 °C water bath using a custom-made restraining device. The animal was positioned in a way so that the tumour faced the center of the ultrasound therapy transducer at a distance calibrated for the maximal focused signal. Prior to initiating ultrasound, animals were injected with 100 µL bolus of microbubble followed by 150 µL of 0.2% heparin–saline flush via a tail-vein catheter. Following microbubble injection, tumour was exposed to ultrasound duration for 5 minutes. The ultrasound system consisted of (AWG520, Tektronix) waveform generator with an (RPR4000, Ritec) amplifier, and a 500-kHz central frequency (Valpey Fisher Inc., MA, USA) transducer (focused at 8.5 cm with a focal point −6 dB beamwidth of 31 mm). The ultrasound parameter used in this experiment was 16 cycles tone burst over 50 msat 500 kHz with a 3 kHz pulse repetition frequency. A delay time of 1950 ms to allow capillary filling was given prior to repeating the pulse sequence. The pulse sequence was continuously repeated over 5-minute duration. A peak negative pressure of approximately 570 kPa corresponding to a mechanical index of approximately 0.8 at the focus was applied.

### Hyperthermia Treatment

HT treatments were administered 5 hours following the ultrasound microbubble treatment. This duration was based on the work^44^ which indicated a treatment interval of 5 hours to be ideal for vascular-related death effect. Custom-made restrainer was used to position the mice with its tumour-bearing leg submerged in 43 **°**C water bath. The duration for HT treatment included from 10 to 60 minutes.

### Data Acquisition and Analysis

Ultrasound RF data was collected before and 24 hours after treatment using a VEVO-770 (Visual Sonics, Toronto, Canada) consisting of a central frequency of 25 MHz and an RMV-710B transducer. A lateral resolution 149 µm and axial resolution 54 µm with approximately 60–100 frames were acquired per scan to obtain RF and B-mode data.

QUS spectral analysis was performed on ultrasound RF data. Ultrasound RF data were collected from 10 to 15 planes from the region of interest of tumour core (approximately 5–10 × 5–10 mm in-plane and 5–10 mm through-plane). Prior to calculating the power spectrum using the Fourier transform, the RF data from individual scan lines were windowed using a Hamming function.^45^ An averaged power spectrum was obtained by averaging power spectra over several independent scan lines within the kernel. The tumour power spectrum was normalized to remove system dependent effects using a reference spectrum that was obtained from a high frequency reference phantom. The speed of sound and attenuation values of our reference phantom are 1540 m/s and 0.556 dB/MHz/cm, respectively.

QUS spectral parameters were obtained from the best-fit line on the normalized power spectrum within a −6 dB window from the transducer center frequency. These parameters included MBF- which is the value of fitted line at the center of the band, SS – the slope of the fitted line, and SI – the interception of the fitted line with 0 MHz axis. These parameters are related to backscatter power, effective scatterer size, and acoustic concentration. All three parameters were determined from each scan plane and averaged over scan planes for each treatment category per tumour. QUS parametric images using a sliding window with approximate size three-pulse lengths were generated for visual display of QUS analysis.

### Histology

Mice were euthanized by cervical dislocation and tumours were excised 24 hours post-ultrasound microbubble and HT treatment. The excised tumour was dissected into half and fixed in 10% neutral buffered formalin for 48 hours at room temperature. Later the specimen was transferred to 70% ethanol. A fixed specimen was then sent to embedding into paraffin blocks. The blocks were further sectioned on glass slides for H&E and TUNEL staining for qualitative analysis of cell morphology and cell death, respectively. All the histology staining was performed in Pathology Research Program, University Health Network, Toronto, ON, Canada.

Low-magnification images of slides were acquired using light microscope and digitalized image files were run through in house developed MATLAB program to evaluate cell death. The average percentage of cell death was calculated from each animals per treatment condition.

### Statistical Analysis

Statistical analysis was performed using Graph Pad Prism software (Graph Pad Software, La Jolla, CA, USA) using analysis of variance (ANOVA) followed by Bonferroni's selected comparisons test. *P*-values (*P* < 0.05) were considered statistically significant and are indicated by asterisk (*). Each treatment group was compared with the controls (untreated group).

## Results

**Figure 1.**
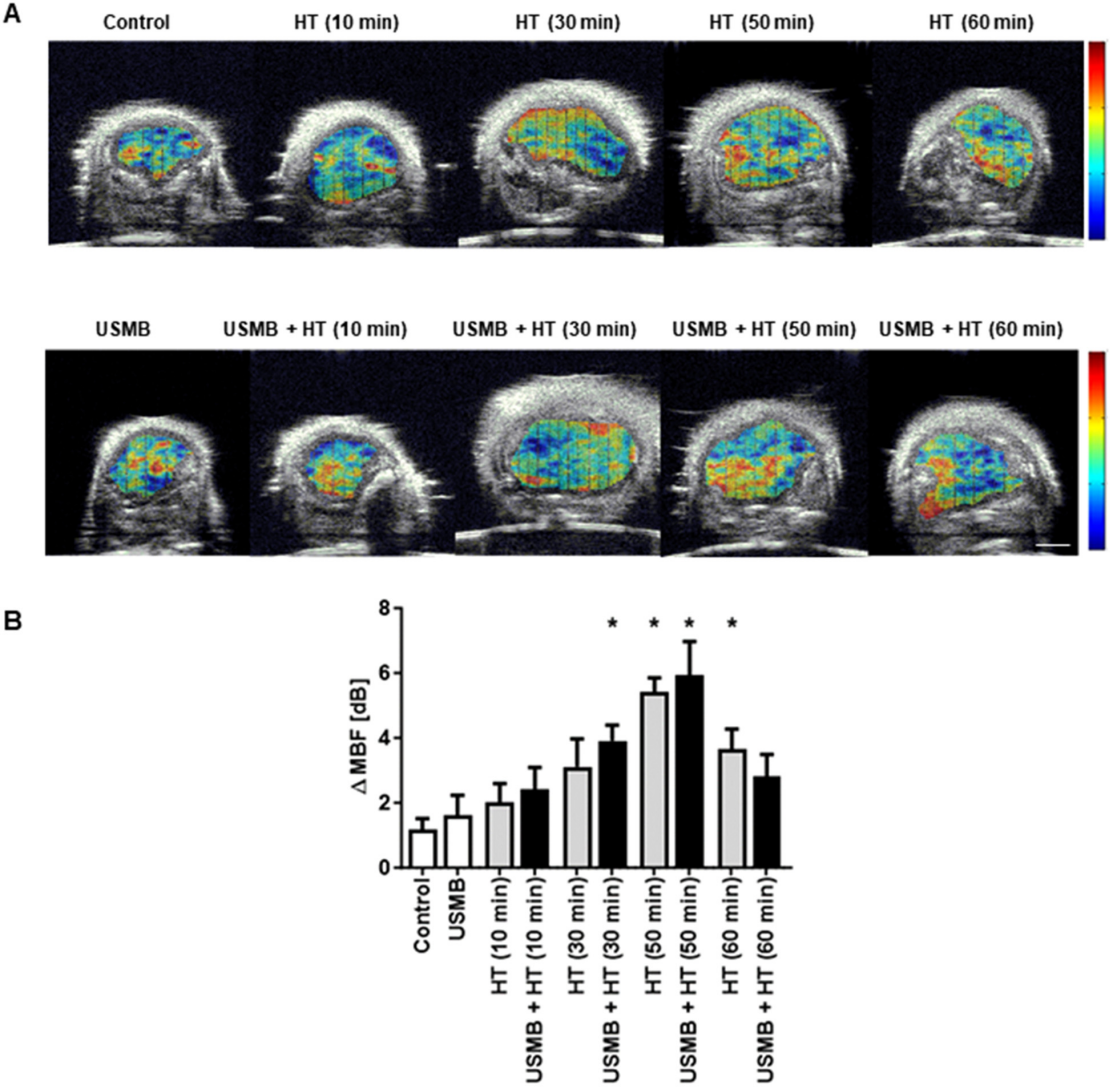
Representative parametric maps of the MBF and changes in the MBF parameters of MDA-MB-231 tumours. (A) Parametric maps of the MBF parameter for breast tumour xenografts. Images are representative of 10 regions of interest per animal. Colour scale represents a range of 20 dBr. The scales bar represents 2 mm. (B) The graph indicates an average change in the MBF parameter. Error bars correspond to the standard error of the mean. All statistical comparisons are compared with the control (untreated) group. Statistical significance was carried out using Graph pad Prism one-way ANOVA followed by Bonferroni selected comparison test. *P* < 0.05 was considered statistically significant and is indicated by asterisks (*). USMB, ultrasound-stimulated microbubbles; HT, hyperthermia; MBF, mid-band fit; ANOVA, analysis of variance.

**Figure 2.**
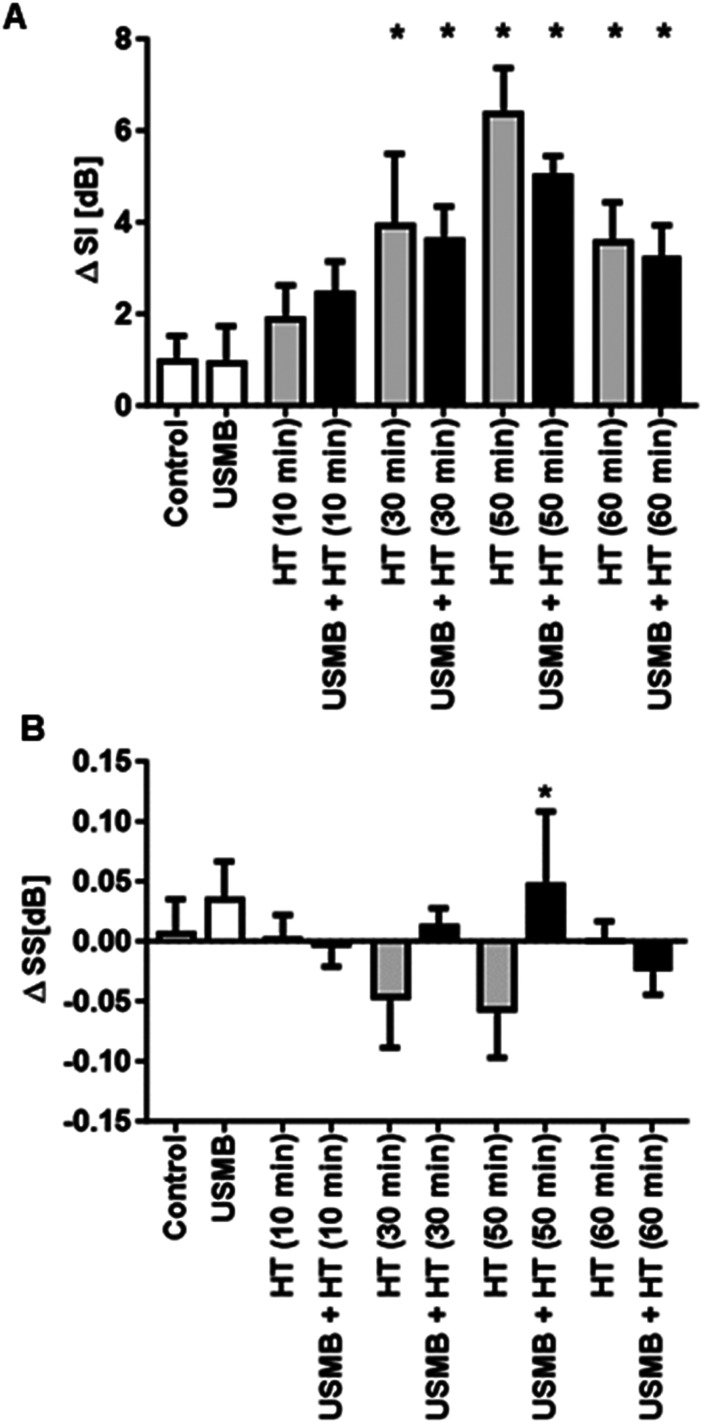
Changes in quantitative ultrasound parameters SI and SS with USMB and HT treatment. (A) Plots of average changes in the SI and (B) SS parameters. Error bars correspond to the standard error of the mean. All statistical comparisons are compared with the control (untreated) group. Statistical significance was carried out using Graph pad Prism one-way ANOVA followed by Bonferroni selected comparison test. *P *< 0.05 was considered statistically significant and are indicated by * asterisks. USMB, ultrasound-stimulated microbubbles; HT, hyperthermia; SI, spectral 0-MHz intercept; SS, spectral slope; ANOVA, analysis of variance.

**Figure 3.**
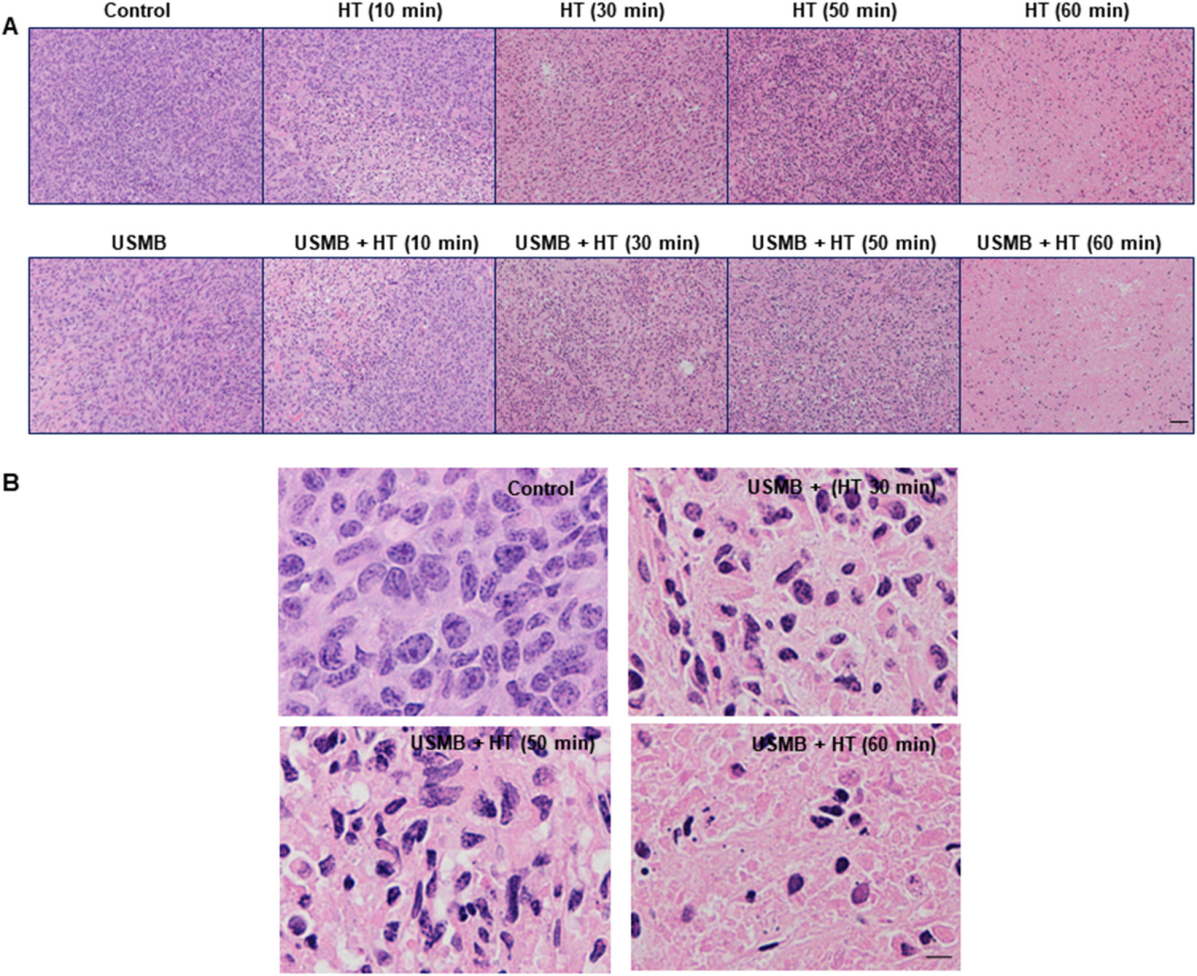
Representative high-magnification (H&E) staining at 24 hours after treatment obtained at (20×) (A). Representative high-magnification images of H&E staining obtained at (60×) (B). Tumours treated with USMB and HT exhibited greater distorted cell morphology compared to the control group demonstrating a higher number of viable cells. With 60 minutes HT duration, most of the cells displayed a lack of nuclei, as a final late phase of apoptosis. The scale bar for images represents 50 µm. USMB, ultrasound-stimulated microbubbles; HT, hyperthermia.

**Figure 4.**
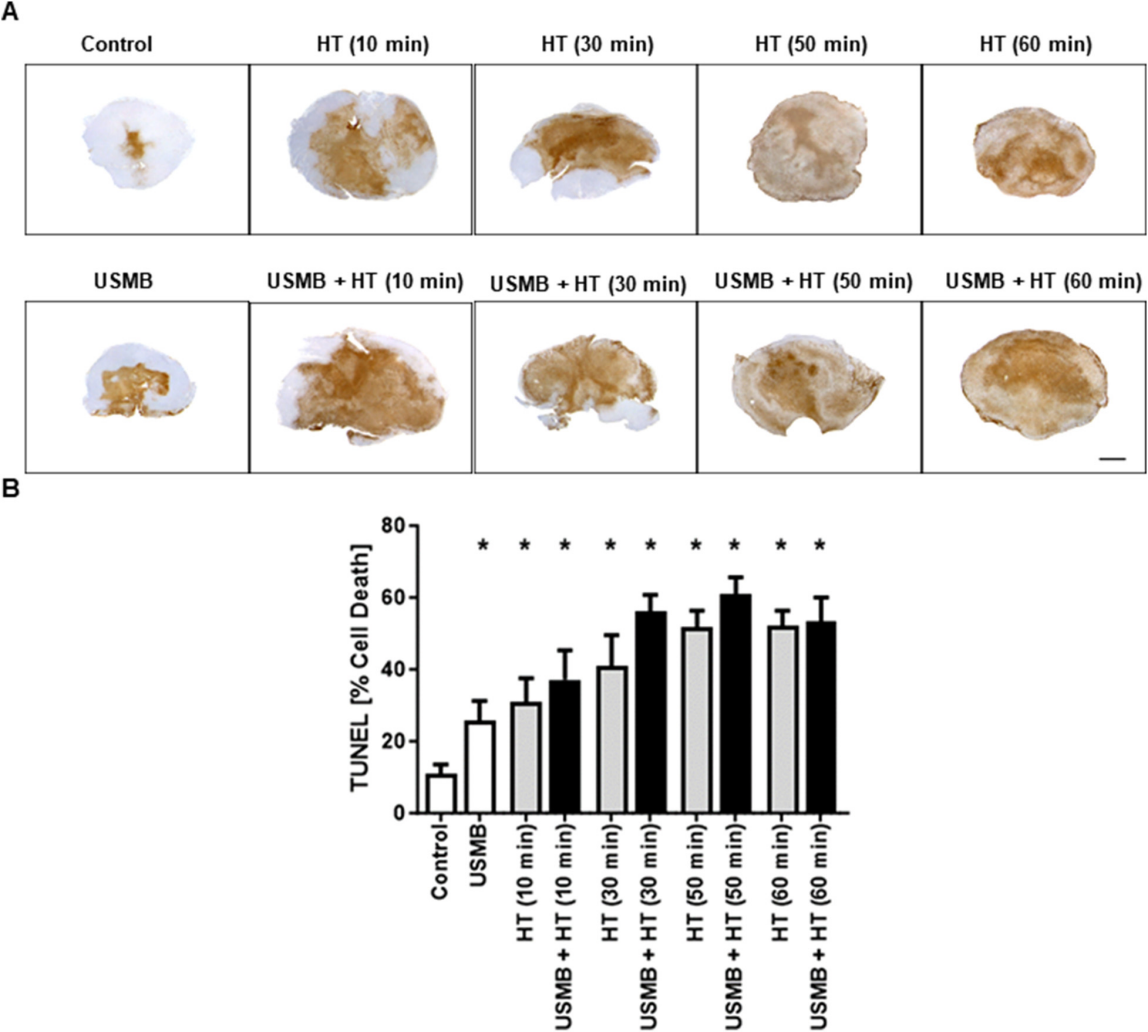
Representative low-magnification (TUNEL) staining at 24 hours after USMB and HT treatment and quantitated tumour cell death. (A) Light microscopy images of TUNEL stained sections taken at low magnification. The scales bar represents 1 mm. (B) Quantification of cell death using low-magnification images. Error bars correspond to the standard error of the mean. All statistical comparisons are compared with the control (untreated) group. Statistical significance was carried out between different groups using a Graph pad Prism one-way ANOVA followed by Bonferroni selected comparison test. Significant differences (*P* < 0.05) are indicated by * asterisks. USMB, ultrasound-stimulated microbubbles; HT, hyperthermia; ANOVA, analysis of variance. Adapted with permission from Sharma, D. et al. (2020) ‘Optimization of microbubble enhancement of hyperthermia for cancer therapy in an in vivo breast tumour model’, PLOS ONE. Edited by S. H. Dairkee, 15(8), p. e0237372. doi: 10.1371/journal.pone.0237372, Figure 4B & C.

Representative parametric images of the MBF obtained from the RF data are presented in Figure 1A. An increase in MBF value was observed in combined USMB and HT-treated groups compared to the control untreated group. Figure 1B represents average changes in the MBF parameter. The MBF values significantly increased with an increase in HT duration. Quantitative analysis demonstrated an average increase in MBF values from 1.16 ± 0.35 dBr for control tumours to 3.91 ± 0.48 dBr and 5.93 ± 1.04 dBr for USMB + 30 minutes HT and USMB + 50 minutes HT-treated tumours, respectively. The MBF values decreased at 60 minutes HT irrespective of USMB addition indicating 3.64 ± 0.63 dBr for 60 minutes HT only and 2.82 ± 0.67 dBr for USMB + 60 minutes HT-treated group. The average change in SI and SS parameters are depicted in Figure 2A and B, respectively. The average changes in SI which corresponds to the concentration of scatterers increased as a function of time after HT treatment and reached its maximum at 50 minutes HT with or without USMB combined. However, a reduction in SI was observed with 60 minutes heating duration, a trend seen homologous to the MBF (Figure 1B). The average change in SS increased only with 50 minutes HT combined with USMB compared to 50 minutes HT alone but remained unaltered with 10, 30, and 60 minutes HT with or without USMB (Figure 2B). Figure 3 illustrates the representative histologic results after USMB and HT treatment. H&E staining obtained at high magnification (20×) demonstrated changes in cell structure in treated areas that increased considerably with HT duration (Figure 3A). To view the alteration in cell morphology more clearly, images were obtained at 60× magnification (Figure 3B). The cells in the control group appeared viable with intact nuclei whereas USMB combined with HT exposed group demonstrated distorted tumour cells with abnormal morphology. Treatment condition consisting of 50 minutes HT alone or combined with USMB exhibited maximum effect. Increasing the HT duration to 60 minutes resulted in the degradation of cell nuclei in a significant manner. More than 50% of the cells in a combined-treated group of 60 minutes HT and USMB showed the absence of nuclei. Representative low-magnification microscopy TUNEL images depicting the gross apoptotic region (brown) with different permutation is shown in Figure 4A. Quantification of cell death from the TUNEL stain tumour section is shown in Figure 4B. An increase in cell death was seen starting 10 minutes and finally plateauing at 50 minutes HT treatment. No further increase in cell death was observed with an increase in HT timing. The combined USMB and 50 minutes HT-treated group displayed a higher percentage of apoptotic cell death reaching 61.06 ± 4.57% (mean ± SEM) compared to 10.88 ± 2.67% for the control untreated group.

## Discussions

In the present study, we aimed to investigate if QUS-based spectral parameters including the MBF, SI, and SS can be used to determine tumour response 24 hours post-USMB and HT treatment. Several studies conducted *in vivo, in vitro*, and *ex vivo* have associated changes in backscattered ultrasound parameters with alteration in tumour morphological characteristics.^24,25,27-29,46,47^ In an *in vitro* study by Czarnota and colleagues, it was shown that apoptotic cells exhibited a twofold higher ultrasound backscattering compared to viable cells. Apoptotic cells demonstrated a higher backscatter intensity at the focal region of the ultrasonic image, contrary to viable cells depicting less intense images. It was suggested that apoptotic cells containing nuclear material in a condensed manner were responsible for increased ultrasound scattering.^48^ A study by Kolios *et al* demonstrated that changes in the spectral parameters (MBF and SS) significantly correlated with the nuclear condensation and fragmentation, considered as a hallmark of apoptosis. Human acute myeloid leukaemia cells (AML-5) upon exposure to (colchicine and DNase) particularly known for causing nuclear condensation or fragmentation resulted in increased MBF and SS.^49^ A similar observation was reported by Banihashemi *et al* demonstrating HTB-67 tumour xenograft treated with photodynamic therapy exhibited a significant increase in the MBF and SS starting 1 to 24 hours which corresponded to higher nuclear coalescence and fragmentation in apoptotic cells. The result further demonstrated a drop in the MBF and SS at 48 hours. Histology stained section obtained at 48 hours revealed majority of the cells lacked their nuclei which were correlated with the diminishment in ultrasound scattering.^29^ Numerous other studies have also confirmed the correlation between ultrasound backscatter and changes in the tissue structural morphology using acute myeloid leukaemia, bladder, breast, and prostate xenograft.^24,27,46,47^ The data from our study here is consistent with the aforementioned findings. We observed a significant increase in ultrasound backscatter parameters (MBF and SI) with an increase in HT duration. Our histology data obtained from treated tumours revealed typical features of apoptosis-like DNA fragmentation, nuclear condensation, the formation of apoptotic bodies, and cell shrinkage. In our study here increasing the HT duration to 60 minutes resulted in a drastic decrease in spectral parameters. When evaluated histologically, a greater population of the cells was found to lose their nuclei which could explain the declination of MBF and SI, a similar phenomenon reported by Banihashemi and colleagues. Studies suggest that ultrasound scattering increases when cells enter the early phase of apoptosis while decrement in scattering is observed as a result of the late apoptotic phase.^29,46^ In those studies, the decrease in backscatter was observed between 24 and 48 hours and 48 and 72 hours. In our study, the decrease in scattering is observed within 50 to 60 minutes of HT treatment which might be a short duration for any obvious changes to occur at the molecular level. However, it is important to consider that a slight difference in HT temperature or duration can impact the morphology of cells and tissue dramatically. Cells exposed to HT can survive to a certain extend acquiring thermotolerance, however, with an increase in heating duration, cells become less resistant to lethal dose and can die by apoptosis or necrosis.^50,51^ Depending on the HT duration, cells can activate several proteins to survive heat, however, extreme heat can cause protein degradation.^52,53^

It is worth noting that most of the studies have reported the changes in the MBF and SS to correspond directly with what is observed histopathologically.^29^ However, in our study, the MBF and SI instead of SS were linked directly with tissue structural changes. The SS parameter remained unaltered except for 50 minutes HT combined with USMB which was found to significantly increase compared to 50 minutes HT group. The rationale for this observation might be due to the effect of HT treatment which works differently than any other cancer treatment modality including radiation therapy or chemotherapy. The effect seen with HT is not only based on the heating duration but also the temperature applied that ranges from mild, moderate, and extreme. A slight increase or decrease in the temperature and duration can have a profound effect on cell and tissue structure.^54,55^ It has been shown that human tissue exhibits five thermal transitions between slight changes in temperature.^56^ Another possibility could be due to the tumour architecture of breast xenograft used in this study. A prior study by Sadeghi-Naini *et al* demonstrated that prostate (PC3) and breast (MDA-MB-231) xenografts, when exposed to chemotherapy exhibited different outcomes in context to SS parameters.^28^ On one hand, where PC3 tumours demonstrated a significant increase in slope parameters, the SS of breast tumours varied insignificantly. The unaltered changes in SS were attributed to be due to the development of different responses by larger scattered structures (glands) in human breast tumours. On the contrary, prostate cancer consists of small stem cell-like tumour cells that are highly organized which might be correlated to the observed increase in slope parameters.^28^

It should be noted that our study here is in agreement with previous studies that show a correlation between QUS biomarkers with cell death suggesting enhanced tumour response either with HT treatment alone or HT combined with USMB. However, tumour response was found to be similar with both the treatment modalities suggesting the lack of synergy between these modalities. It seems that the potency of the HT treatment alone can cause significant tumour cell death subsequently increasing QUS backscatters wherein the addition of USMB to the HT group increased the tumour effect in a non-significant manner.

The limitations of this study are: firstly, the use of conventional HT technique using a water bath. With this form of HT, we can only determine the temperature and heating duration; however, the energy dose deposited cannot be evaluated. Secondly, the biological and molecular mechanisms behind cell death were not evaluated in this study. Knowing the mediator that triggers the stress within a cell might help understand the correlation between QUS biomarkers with the extent of tumour cell death in a better way.

Future studies should incorporate heat delivery using magnetic HT or modulated electro-HT that specifically targets a focal area of tumours. The QUS results obtained from this form of HT should be compared with conventional HT results to see if the treatment outcome is improved. Next, the duration between USMB and HT should be explored further. In this study, no differences in tumour response were seen between HT alone or USMB combined with HT. A shorter duration of <10 minutes between both these modalities should be assessed to see if the tumour response changes from what is obtained here. Furthermore, the effects of multiple treatments in longitudinal studies should be optimized and assessed on larger tumours model or in orthotopic tumours.

## Conclusion

In this study, we attempt to explore whether or not a correlation exists between ultrasound spectra and histological estimates of cell death following treatment of USMB and HT in the breast xenograft model. Results demonstrated an increase in the QUS backscatter parameters (MBF and SI) with an increase in HT duration till 50 minutes that correlated with significant changes in cells and tissue structure confirmed using histopathologic assessment. However, at 60 minutes HT, both the parameters significantly reduced as a result of vanishing nuclei. The slope parameters remained unchanged irrespective of HT duration. In conclusion, our study supports the finding from previous studies suggesting QUS techniques may be utilized to assess cell death *in vivo*.
